# Differential Effect of Level of Self-Regulation and Mindfulness Training on Coping Strategies Used by University Students

**DOI:** 10.3390/ijerph15102230

**Published:** 2018-10-11

**Authors:** Jesús de la Fuente, Israel Mañas, Clemente Franco, Adolfo J. Cangas, Encarnación Soriano

**Affiliations:** 1 School of Education and Psychology, University of Navarra, Campus Universitario s/n. 31009 Pamplona, Spain; jdlfuente@unav.es; 2 School of Psychology, Universidad Autónoma de Chile, Avenida Pedro de Valdivia, 425, Providencia, Santiago de Chile, Chile; 3 Department of Psychology, University of Almería, Cañada de San Urbano s/n. 04120 Almería, Spain; cfranco@ual.es (C.F.); ajcangas@ual.es (A.J.C.); 4 Faculty of Psychology and Education Sciences, Universitat Oberta de Catalunya, Rambla del Poblenou, 156, 08018 Barcelona, Spain; 5 Department of Education, University of Almería, Cañada de San Urbano s/n. 04120 Almería, Spain; esoriano@ual.es

**Keywords:** self-regulation, mindfulness, coping strategies, students, higher education

## Abstract

The purpose of this research was to verify, in a group of psychology students, whether mindfulness training in conjunction with the individual’s level of self-regulation behavior would produce a change in the use of coping strategies. A total of 38 students participated in this study, with one experimental group and one control group, in a randomized controlled trial. Observation of the experimental group revealed a significant decrease in specific emotion-focused, negative coping strategies (preparing for the worst, resigned acceptance, emotional venting, and isolation), and a significant increase in specific problem-focused, positive coping (positive reappraisal and firmness, self-talk, help for action), in combination with students’ existing low-medium-high level of self-regulation. The importance and usefulness of mindfulness techniques in Higher Education is discussed, in conjunction with differences in university students’ level of self-regulation behavior.

## 1. Introduction

Within the sphere of psychology research that examines stress in academic environments, it is especially important to analyze differences in the use of motivational-affective strategies. In recent years, interest has expanded beyond understanding meta-cognitive strategies used while learning to knowing how motivational-affective processes are regulated and how the latter affect and interact with the former [1]. One example of this is the important role that is ascribed to students’ self-regulated behavior and to the regulatory characteristics of the context.

### 1.1. Self-Regulation as a Meta-Behavioral Variable

The Theory of Self vs. Externally-Regulated Learning [2] considers Self-Regulation (SR) to be a meta-behavioral skill that predicts numerous student behaviors; three possible behavior types are established in interaction with the context (self-regulation, a-regulation, and dysregulation). Previous research has described Self-Regulation as multidimensional, referring to one’s level of effort in becoming actively engaged in behavior to accomplish a task. It is a psychological construct that has acquired a great deal of research relevance, given its verified relationship to improvement of the individual’s health, well-being, and academic, personal, and professional success [3,4,5,6]. Several studies on personal self-regulation and psychological well-being have shown that students in higher education with self-regulation skills experienced fewer problems with addictive behaviors (e.g., alcohol) than those lacking self-regulation skills [7,8,9]. They also showed better social adaptation [10] and academic achievement [11]. 

### 1.2. Mindfulness-Based Interventions

The term mindfulness can be used to describe a theoretical construct (mindfulness), a practice of cultivating mindfulness (meditation), or a psychological process (being mindful) [12]. An often-cited definition of mindfulness is “paying attention in a particular way: on purpose, in the present moment, and nonjudgmentally” [13] (p. 4). Another frequent definition is “the nonjudgmental observation of the ongoing stream of internal and external stimuli as they arise” [14] (p. 125). Broadly conceptualized, mindfulness has been described as a kind of nonelaborative, nonjudgmental, present-centered awareness in which each thought, feeling, or sensation that arises in the attentional field is acknowledged and accepted as it is [15].

With the intent to develop an operational definition, Bishop et al. [15] proposed that mindfulness encompasses two components: (1) self-regulation of attention, referring to non-elaborative observation and awareness of sensations, thoughts, or feelings from moment to moment; and (2) adoption of a particular orientation towards one’s experiences; a kind of attitude that one holds towards one’s experience, specifically an attitude of curiosity, openness, and acceptance. From the mindfulness perspective, acceptance refers to a willingness to let things be just as they are at the moment we become aware of them and accepting pleasurable and painful experiences as they arise [12]. 

In the past three decades, a great number of mindfulness-based interventions (MBIs) have become increasingly popular (for a recent and exhaustive review see Creswell, 2017). Germer [12] proposed four leading MBIs: (1) Dialectical Behavior Therapy (DBT) [16], which has become the preferred treatment for borderline personality disorder and is being used for affect regulation in general. Mindfulness skills in DBT are considered core skills for success: interpersonal effectiveness, emotion regulation, and tolerance of distress; (2) Mindfulness-Based Stress Reduction (MBSR) [17], which is an 8- to 10-week mindfulness training course with multiple applications to physical and mental health. The main practices taught are sitting meditation (mindfulness breathing), mindful yoga, body scan meditation, and mindfulness in everyday life, including walking, standing, and eating; (3) Mindfulness Based Cognitive Therapy (MBCT) [18], which is an application of MBSR to cognitive therapy and depression, and teaches patients to observe their thoughts. MBCT teaches the mindfulness practices of MBSR, without the yoga, and with the three-minute breathing space exercise; (4) Acceptance and Commitment Therapy (ACT) [19] encourages patients to accept, rather than control, unpleasant sensations. ACT uses metaphors, paradoxes, experiential exercises, cognitive defusion techniques, and mindfulness.

In addition to these four MBIs, the recent mindfulness training program “Flow Meditation” (FM) should also be noted [20,21,22,23,24]; its primary objective is not to try and control thoughts, sensations, or feelings or to modify or change them, but conversely, to allow them to be free, accepting any private event that might occur or emerge spontaneously. FM employs mindfulness breathing, body scan meditation from MBSR, and metaphors and exercises from ACT. 

MBIs have proven to be effective in psychological distress, physical health (e.g., chronic pain, immune system, and many diseases and illnesses like cancer or fibromyalgia), mental health (e.g., depression, anxiety, addiction), improving cognitive functioning (e.g., attention, concentration, memory), emotional and behavioral regulation, well-being, quality of life, and interpersonal relations, including healthy populations and individuals with mental disorders [25,26,27]. Extensive descriptions of MBIs as well as their underlying mechanisms of change can be found elsewhere [14,28,29].

### 1.3. Coping Strategies as a Meta-Emotional Variable

Coping strategies refer to a constellation of strategies that healthy people apply in problem solving. Several reviews indicate a strong association between the experience of stressors and the presence of emotional and behavioral problems [30]. However, despite this evidence, it has been suggested that psychological well-being and health are more influenced by the manner of coping with stress than by the mere presence of difficult situations [31,32]. 

According to the classic transactional model of coping proposed by Lazarus and Folkman [33], distress occurs when a person perceives that environmental demands exceed their capacities and available resources and, on this basis, they make an appraisal of the stressful factor, thereby determining the level of stress that they experience. “Psychological stress is a particular relationship between the person and the environment, that is appraised by the person as exceeding or taxing his or her resources and endangering his or her well-being” [34] (p. 19).

Coping refers to “cognitive and behavioral efforts to master, reduce, or tolerate the internal and/or external demands that are created by the stressful transaction” [33] (p. 843). Given the existing diversity of responses to stress, most authors have tried to make significant, meaningful categorizations (e.g., active versus passive or avoidant coping). Lazarus and Folkman proposed that coping serves two major functions. One is the regulation of emotions or distress that come with the stressful situation (emotion-focused coping). The other is management of the problem that is causing the stress by directly changing the elements of the stressful situation (problem-focused coping). Although both forms of coping are used in most stressful encounters, they are nevertheless dependent on the way one appraises the situation (i.e., as a threat and/or a challenge) and on its antecedents. Lazarus and Folkman identified at least two broad categories of antecedents which will directly influence how people appraise and cope with situations: those linked to characteristics of the individual, and those linked to characteristics of the situation. In the first category, for example, we find commitments (which define what is important to the person and, therefore, what is at stake in the situation), their beliefs, such as beliefs about personal control, and so on. Among situational factors, we find the novelty vs. predictability of the situation, the uncertainty of the event, time factors (time generally enhances the threat, however there may also be time to think something through), and the ambiguity of the situation.

Strategies represent an effort toward modifying stressor factors, whether attributed to the individual or the environment [35]. The first group of such coping strategies comes under the umbrella of problem solving or directly taking action to confront problems. The second group includes social support, situational change, and psychological adjustment. A third group from the literature review relates to emotional avoidance, an individual orientation toward giving up without struggling for control of the conflictive situation, or just accepting with resignation.

### 1.4. Self-Regulation, Mindfulness and Coping Strategies

MBIs directly or indirectly include training in emotion regulation skills. In fact, the most important therapeutic component of DBT is precisely its training in emotion regulation skills. In a less direct manner, ACT, MBCT, MBSR and FM also incorporate the general objective of acquiring and exercising emotion regulation skills, including development of more effective, healthier coping strategies.

Several studies show the beneficial effects of mindfulness on different processes of emotion regulation. Arch and Craske [36] showed that a group of participants who were instructed to practice 15 min of mindfulness breathing responded positively to neutral images, in comparison to two other groups—one group who was instructed to not focus their attention, and another was induced toward worry—who responded with negative emotional reactions. The authors suggest that focusing attention on breathing acts as a key mindfulness mechanism for emotion regulation. Using a sample of psychology students, Evans, Baer, and Segerstrom [37] found that mindfulness, particularly its facets of not reacting and not condemning, led to increased persistence on laboratory tasks with a certain degree of difficulty, through emotion self-regulation processes. More recently, Hülsheger, Alberts, Feinholdt, and Lang [38], with a sample of workers from different organizations (hospitals, schools, shops, public offices, etc.), found that mindfulness produced significant increases in emotion regulation and job satisfaction, and a decrease in emotional fatigue. Finally, Brockman, Ciarrochi, Parker, and Kashdan [39] examined the effects of three emotion regulation strategies (emotion suppression, cognitive reappraisal, and mindfulness) on the experience of daily negative and positive affect in college students. Their results indicated that emotion suppression was related to higher negative and lower positive affect, that cognitive reappraisal was associated with positive, but not negative affect, and that mindfulness produced lower negative and higher positive affect.

As for coping strategies, research studies show that mindfulness promotes and improves the use of a broad and varied range of positive coping strategies. Weinstein, Brown, and Ryan [40], with a sample of psychology students, found that individuals with higher levels of mindfulness made more benign attributions about stress, showed less use of avoidant coping, and greater use of approach coping. Kang, Choi, and Ryu [41] examined the effectiveness of a stress coping program, based on mindfulness meditation, on the stress, anxiety, and depression that was experienced by nursing students. Results showed that participants in the experimental group made significant improvements in adaptive coping strategies and in the variables of stress and anxiety, however no effect was found on the variable depression. More recently, Taylor et al. [42] examined several ways by which mindfulness reduced teacher stress. In essence, results showed a trend for teachers from the experimental group to report more adaptive strategies for coping with job stress and a tendency to evaluate challenging students in a more positive affective light. Finally, Pidgeon and Pickett [43] examined differences in psychological distress, mindfulness, and coping strategies (adaptive vs. maladaptive) in university students with high and low levels of resilience. The results showed that the university students with a higher level of resilience reported significantly higher levels of mindfulness, greater use of adaptive coping strategies, reduced maladaptive coping, and lower levels of psychological stress, when compared to students with low resilience levels.

### 1.5. Objectives and Hypotheses

Despite plentiful prior knowledge of the strengths of mindfulness as a technique for emotion regulation, there is still not a precise understanding of how it affects students differently according to their different existing levels of personal self-regulation, in other words, if self-regulation is a mediating variable in the effects of mindfulness treatment. Therefore, the objective of this study was to determine how a training program in mindfulness interacts with university students’ prior levels of self-regulation with the use of different types of stress coping strategies. Based on prior evidence and the existing conceptual relationships, the following hypotheses were posed:

1. In general, the students’ level of self-regulation would be interactive with mindfulness treatment. Specifically, a greater level of prior personal self-regulation should be accompanied by lesser use of inadequate emotion-focused strategies and an increase in adequate emotion-focused strategies, as well as maintenance of problem-focused strategies. In contrast, students with a lower level of self-regulation would increase their use of emotion-focused strategies, however not problem-focused strategies.
2. More specifically, a significant increase is expected in coping behaviors that are typical of mindfulness, in accordance with students’ level of self-regulation and the treatment received. Thus, it would seem plausible to find that, with greater self-regulation, there would be a decrease in strategies that represent a negative handling of emotion (suppressing feelings, avoidance, and substance use), an increase in positive strategies for handling emotion (expressing feelings, positive attitude), and a greater number of problem-focused strategies (help-seeking and social support).

## 2. Materials and Methods

### 2.1. Participants

The sample was composed of 38 students from the University of Almeria (Spain) who were enrolled in the Faculty of Psychology; ages ranged from 18 to 29 years (*M* = 24.36; *SD* = 4.72). The experimental group was formed of 19 students (2 male and 17 female), and the control group contained another 19 students (3 male and 16 female). Subjects were assigned to one group or the other on a random basis, controlling for the gender variable so that there would be a similar number of men and women in both groups and, thus, avoiding possible interference from this variable in our results. The variable of year in school was also controlled, such that there were a similar number of students from different stages in their degree in each group to keep this variable from contaminating the research results.

### 2.2. Instruments

Self-Regulation Questionnaire, SRQ [44]. The questionnaire used for this research was the abridged version of the original questionnaire on self-regulation, which was developed by the authors to measure self-regulation of behavior in general, defined as what leads people to plan and flexibly address their own behavior according to the demands of the environment, through a series of strategies [45]. Pichardo et al. [46] validated the Spanish Self-Regulation Questionnaire, Short version, SSSRQ, a reduced version of 17 items on a 5-point Likert scale ranging from 1 (not at all) to 5 (very much). Four dimensions were obtained by both exploratory and confirmatory factor analysis. The confirmatory factor analysis that was performed for this sample showed the four dimensions (Goal setting, Perseverance, Decision-making, and Learning from mistakes). The authors reported a total reliability value of alpha = 0.87, and subvalues for F1 (alpha = 0.81), F2 (alpha = 0.71), F3 (alpha = 0.76), and F4 (alpha = 0.79), with adequate general values, despite having better adjustment in the case of the female students. More recently, a revalidation using rasch methodology has been reported, with similar, consistent results [31].

Coping Strategies Scale. The EEC [47] was used in a short, validated Spanish version, EEC-Short [48]. Although the original instrument contained 90 items, the validation produced a first-order structure of 64 items and a second order with 10 factors and two dimensions, both of them significant, with adequate fit values in the latter case (Chi-square = 878.750; Degrees of freedom (77 − 34) = 43, *p* < 0.001; NFI = 0.901; RFI = 0.945; IFI = 0.903, TLI = 0.951, CFI = 0.903, RMSEA= 0.07). Reliability measurements are Cronbach alpha of 0.93 (complete scale), 0.93 (first half), and 0.90 (second half), Spearman-Brown of 0.84, and Guttman of 0.80. Two dimensions were evaluated: D1—Emotion-focused coping (0.95); D2—Problem-focused coping (0.91). In relation to emotion-focused strategies, the factors were: F1—Avoidant distraction (0.79); F7—Reducing anxiety and avoidance (0.88); F8—Preparing for the worst (0.80); F9—Emotional venting and isolation (0.91); and F10—Resigned acceptance (0.86). In relation to problem-focused strategies, the factors were: F2—Seeking family help and counsel (0.92); F5—Self-talk (0.82); F10—Positive reappraisal and firmness (0.87); F12—Communicating feelings and social support (0.89); and F13—Seeking alternative reinforcements (0.80). See Chart 1.

### 2.3. Procedure

The entire experiment was conducted in accordance with the Declaration of Helsinki of 1975, revised in 2008. All participants also gave oral informed consent. Ethics approval was obtained from the Research Ethics Committee of the University of Almería, Spain (UALBIO2018/020).

First, a course was made available to Psychology students at the University of Almeria (Spain), entitled “Learning and practicing mindfulness”; this served to obtain a study sample. A total of 41 students enrolled in the course, of which 38 later became part of the research study. Students who reported any prior experience with a meditation technique were not taken into account for this study. Once the study sample was formed, the questionnaires were administered to all of the participants for individual completion, thereby obtaining pretest measures for the dimensions of the variables self-regulation and coping strategies. After the pretest score was obtained, subjects were randomly assigned to the control group or experimental group.

Next, the intervention program was applied to the experimental group over 10 sessions (see Chart 2), which were 1½ h sessions that were held weekly. The intervention program consisted of learning and practicing a mindfulness technique for 40 min each day [20,49]. Its main objective was not to try to control one’s breathing, thoughts, physical sensations, or feelings, or to modify or exchange them for others; on the contrary, it was to let them be free, accepting any private event that might appear or spontaneously arise.

Mindfulness breathing is one of the main components of FM. This practice consists of mentally repeating a nonsense word formed by three syllables, while directing attention toward the abdomen and noticing how air goes in and out while breathing, however trying not to change or alter the respiration itself. The objective is awareness of how it happens naturally and without effort. This way, the mind becomes quiet, achieving a state of mental calm and elevated attention and concentration, therefore decreasing automatic and involuntary activity [50].

The objective of FM is to be aware, passively and without effort, of what occurs in our mind and our body, however without trying to make any effort to change or modify it, perceiving things as they are and as they take place each moment. With practice, one develops the ability to observe mental processes (e.g., thoughts, images) and emotional processes, but without getting involved in those processes, and without analyzing them, judging, or controlling them, thus breaking the habit of being led away by thoughts and emotions that are automatic and uncontrolled. In this way, we experience how thoughts, physical sensations, and feelings are changing at every moment and are constantly in flow. In this way, one learns to be present, with an attitude of openness and acceptance in the face of any mental, physical, or emotional process that might occur.

In each of the 10 sessions, besides learning and practicing the mindfulness technique, we made use of different metaphors and exercises from Acceptance and Commitment Therapy, ACT [19]. The objective of these metaphors and exercises was to comprehend and to experience that when we try to control and/or eliminate bothersome and unpleasant private events (e.g., thoughts, feelings, body sensations, etc.), they often increase in frequency, intensity, and duration, further aggravating one’s psychological distress and suffering [51], and therefore, the best option in these cases is to become aware of them, accept them just as they appear, and let them flow freely.

Another component of the mindfulness program, which was also learned and practiced over the 10 sessions, was body-scan exercises [17]. Body scan is a technique where attention is directed to different parts of the body in an ordered, systematic manner, without making any kind of judgment or appraisal, and without trying to change, control, or eliminate anything (e.g., body sensations, mental and/or emotional reactions, etc.). Through this technique, the individual observes how body sensations are continuously changing and learns to not react to them, thereby developing an attitude of acceptance toward these sensations.

Once the mindfulness course was completed in the experimental group, both groups were reassessed with regard to the different dimensions of coping strategies under the same conditions as in the pretest phase. It should be noted that the pretest assessment took place in the month of March, while the posttest assessment was carried out in June, coinciding with the university’s final exam period.

After the investigation was completed, mindfulness training was administered to the control group. All of the study participants were informed regarding the research objective at the end of the study and were asked to give their written consent to make use of the data collected, maintaining and ensuring confidentiality and anonymity.

### 2.4. Data Analysis

A randomized controlled trial was used to analyze the effects of personal self-regulation and mindfulness treatment (IVs) on students’ coping strategies (DVs), where an experimental group and a nonequivalent control group were compared using pre- and post-test measures. Using cluster analysis, the independent variable personal self-regulation was manipulated by selection into high, medium, and low levels. We carried out two Analyses of Variance, univariate and multivariate, Self-Regulation × Mindfulness Treatment × Time, to test the hypotheses, with the following dependent variables: (1) total scores, (2) the two dimensions of coping strategies, and (3) specific coping behaviors. The software package SPSS v. 23 (IBM, New York, NY, USA) was used for these analyses.

## 3. Results

### 3.1. Effect of Self-Regulation Level (SR) × Mindfulness Treatment (M) × Time (T) on Total Coping Strategies

**Figure 1:**
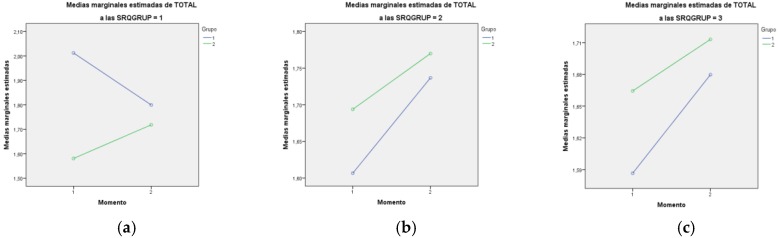
Combined effect of Self-Regulation Level (SRQGRUP)) × Mindfulness Treatment (GRUPS: 1,2) × Time (Moment) on *total coping strategies*. Key: Medias marginales estimadas—estimated marginal means; Momento—time; Grupo—group. (**a**) Effect on the low group of self-regulation; (**b**) Effect on the average group of self-regulation; (**c**) Effect on the high group of self-regulation.

The pertinent ANOVA showed a main effect with a significant interaction of *level of self-regulation (SR)* × *Time (T)* (*F*(2,70) = 4.133 (Pillai), *p* < 0.05, *n*^2^ = 0.123, observed power = 0.709). Thus, while students with low self-regulation did not show a significant increase in the total strategies assessed at each moment, this increase was found in students with medium and high levels. There was no statistically significant main effect of mindfulness treatment on total coping strategies. See specific results in Table 1 and the interaction effect in Figure 1.

### 3.2. Effect of Self-Regulation Level (SR) × Mindfulness Treatment (M) × Time (T) on the Dimensions of Coping Strategies

**Figure 2:**
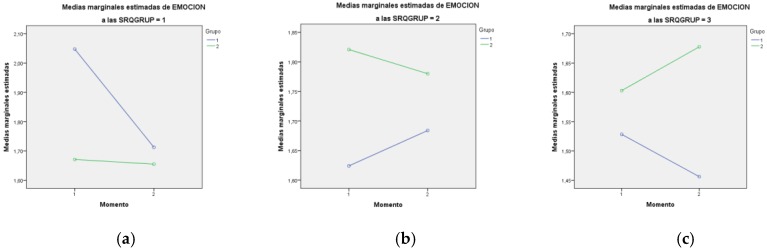
Combined effect of Self-Regulation Level (SRQGRUP) × Mindfulness Treatment (GRUPS) × Time (Momento) on the *emotion-focused dimension*. SR Group by level: 1—low; 2—medium; 3—high. Group: 1—Experimental; 2—Control. Key: Medias marginales estimadas—estimated marginal means; Emoción—emotion; Momento—time; Grupo—group. (**a**) Effect on the low group of self-regulation; (**b**) Effect on the medium group of self-regulation; (**c**) Effect on the high group of self-regulation.

The multivariate analysis SR × M × T showed a significant main effect of the factor Self-Regulation Level (SR) (F(4,118) = 4.389, *p* < 0.001, *n*^2^ = 0.130; observed power = 0.927), with a significant partial effect on Dimension 1, emotion-focused coping (F(2,223) = 6.629 (Pillai), *p* < 0.01, *n*^2^ = 0.183; power = 0.889; post-hoc: 1,2 > 3, *p* < 0.001). It also showed a main effect of the factor mindfulness treatment (F(2,58) = 5.751, *p* < 0.06, *n*^2^ = 0.090; power = 0.542), with a significant partial effect on Dimension 2, problem-focused coping (F(1,70) = 3.3996, *p* < 0.05, *n*^2^ = 0.063; power = 669). Similarly, a main effect of the factor Time was also observed (F(1,70) = 5.945, *p* < 0.01, *n*^2^ = 0.092; power = 0.669), with a significant partial effect on Dimension 2, problem-focused coping (F(1,70) = 3.3996, *p* < 0.05, *n*^2^ = 0.063; power = 669). Finally, a statistically significant main effect was also observed for the interaction SR × M (F(4,118) = 2.173, *p* < 0.06, *n*^2^ = 0.069; power = 0.626), with a partial effect on Dimension 1, emotion-focused coping (F(1,70) = 4.436, *p* < 0.01, *n*^2^ = 0.128; power = 0.732). Specific values are presented in Table 2 and Figure 2. Note how the effect of mindfulness treatment was a decrease in emotion-focused coping strategies in the group of students with low self-regulation, while the medium group increased a bit, and the high group showed a decrease to the lowest level.

### 3.3. Effects of Self-Regulation Level × Mindfulness Treatment × Time on Factors of Emotion-focused Coping

**Figure 3:**
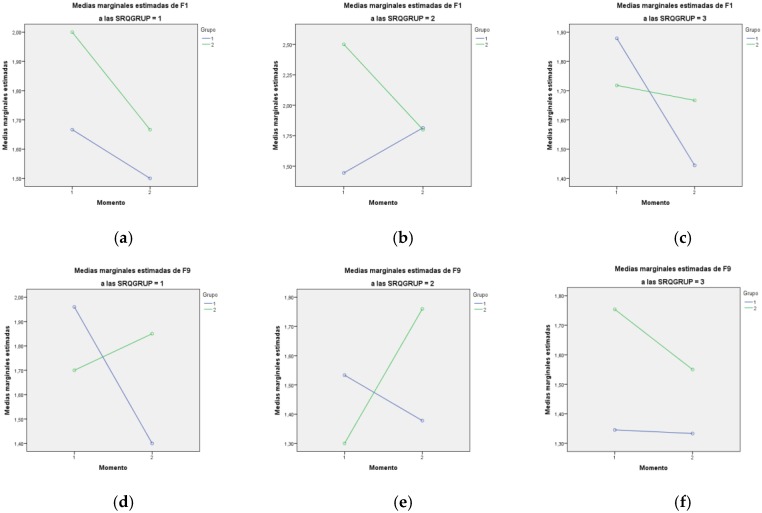
Interaction effect SR (SRQGRUP) × Tratament (GRUPS) × Time (momento) on factors: F1—avoidant distraction; and F9—emotional venting and isolation. SR Group by level: 1—low, 2—medium, 3—high. Group: 1—Experimental; 2—Control. Key: Medias marginales estimadas—estimated marginal means; Momento—time; Grupo—group. (**a**,**d**) Effect on the low group of self-regulation; (**b**,**e**) Effect on the medium group of self-regulation; (**c**,**f**) Effect on the high group of self-regulation.

The multivariate analysis of SR × M × T showed a significant main effect of the factor Self-Regulation Level (SR) (F(12,110) = 2.574, *p* < 0.01, *n*^2^ = 0.219; observed power = 0.967), with a significant partial effect on factor 7, reducing anxiety and avoidance (F(2,70) = 9.956 (Pillai), *p* < 0.001, *n*^2^ = 0.245; power = 0.976; post-hoc: 1, 2 > 3, *p* < 0.001). A main effect was also observed for the factor Treatment (F(6,54) = 4.239, *p* < 0.001, *n*^2^ = 0.219; power = 0.966), with a significant partial effect on Factor 1, avoidant distraction (F(1.70) = 5.835, *p* < 0.01, *n*^2^ = 0.090; power = 0.661) and on Factor 7, reducing anxiety (F(1,70) = 6.206, *p* < 0.01, *n*^2^ = 0.095; power = 688). Similarly, a main effect was observed for the factor Time (F(6,54) = 2.445, *p* < 0.05, *n*^2^ = 0.214; power = 0.781), with a significant partial effect on Factor 1, avoidant distraction (F(1,70) = 3.933, *p* < 0.05, *n*^2^ = 0.064; power = 0.496; 2 < 1). Finally, a significant interaction effect was observed, SR × M [F(12,110) = 1.751, *p* < 0.06, *n*^2^ = 0.160; power = 0.847], with a partial effect on Factor 8, preparing for the worst (F(1,70) = 3.547, *p* < 0.01, *n*^2^ = 0.107; power = 0.838], as well as a significant interaction effect, SR × M × T (F(12,110) = 4.540, *p* < 0.01, *n*^2^ = 0.169; power = 0.875], with a partial effect on Factor 1, avoidant distraction (F(2,71) = 1.868, *p* < 0.05, *n*^2^ = 0.133; power = 0.752] and on Factor 9, emotional venting and isolation (F(2,71) = 1.868, *p* < 0.084, *n*^2^ = 0.518; power = 0.518). Specific values are presented in Table 3 and Figure 3.

### 3.4. Effects of Self-Regulation Level × Mindfulness Treatment × Time on Problem-Focused Factors

**Figure 4:**
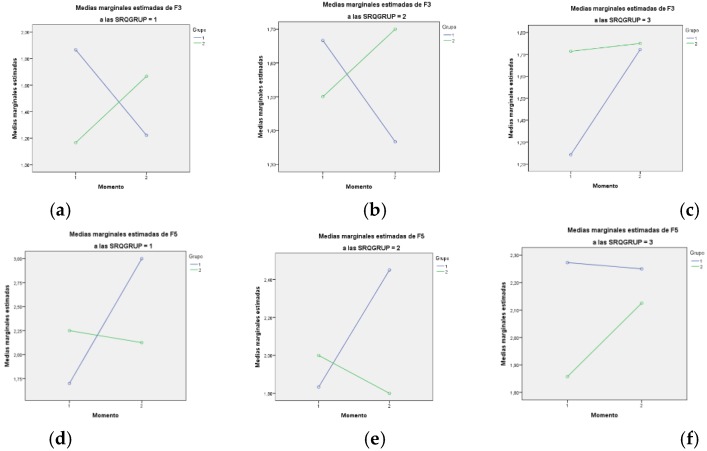

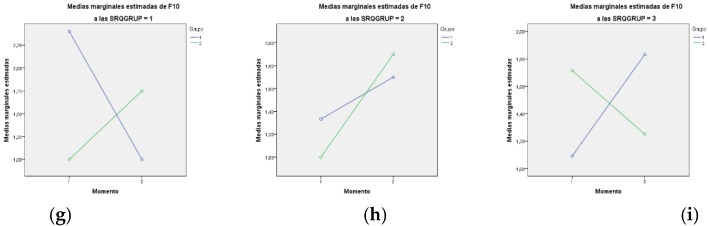
Effect of the interaction SR (SRQGRUP) × Tratamiento (GRUPS) × Time (Momento) on factors: F3—Actions directed at causes; F6—Seeking help for action; and F10—positive reappraisal and firmness. Note: SR Group by level: 1—low; 2—medium; 3—high. Group: 1—Experimental; 2—Control. Key: Medias marginales estimadas—estimated marginal means; Momento—time; Grupo—group. (**a**,**d**,**g**) Effect on the low group of self-regulation; (**b**,**e**,**h**) Effect on the medium group of self-regulation; (**c**,**f**,**i**) Effect on the high group of self-regulation.

The multivariate analysis *SR* × *M* × *T* showed a nonsignificant main effect of *Self-Regulation Level (SR)* (*F*(10,118) = 0.696, *p* < 0.727, *n*^2^ = 0.056; observed power = 0.248), with no effect on any factor. A marginally significant main effect also appeared for *Treatment* (*F*(5,58) = 2.157, *p* < 0.07, *n*^2^ = 0.157; power = 0.668), with a significant partial effect on Factor 13, alternative reinforcement, in favor of the experimental group (*F*(1,73) = 8.513, *p* < 0.001, *n*^2^ = 0.121; power = 0.819). Similarly, a significant main effect appeared for the factor *Time* (*F*(5,58) = 2.412, *p* < 0.05, *n*^2^ = 0.172; power = 0.725), with a significant partial effect on Factor 5, self-talk (*F*(1,73) = 5.326, *p* < 0.05, *n*^2^ = 0.079; power = 0.623; time 2 > 1]. Finally, there was a significant interaction effect, *M* × *T* (*F*(5,58) = 2.476, *p* < 0.05, *n*^2^ = 0.176; power = 0.738], with a partial effect on Factor 5, self-talk (*F*(1,73) = 1.315, *p* < 0.01, *n*^2^ = 0.088; power = 0.675), as well as a significant interaction effect, *SR* × *M* × *T* (*F*(10,118) = 2.231, *p* < 0.05, *n*^2^ = 0.159; power = 0.902], with a partial effect on Factor 3, Actions directed at causes (*F*(2,62) = 3.861, *p* < 0.05, *n*^2^ = 0.111; power = 0.697), Factor 5, self-talk (*F*(2,62) = 4.138, *p* < 0.05, *n*^2^ = 0.118; power = 0.711), and Factor 10, positive reappraisal and firmness (*F*(2,62) = 6.867, *p* < 0.001, *n*^2^ = 0.181; power = 0.909). Specific values are presented in Table 4 and Figure 4.

### 3.5. Effects of Self-Regulation × Mindfulness Treatment × Time on Specific Coping Behaviors

**Figure 5:**
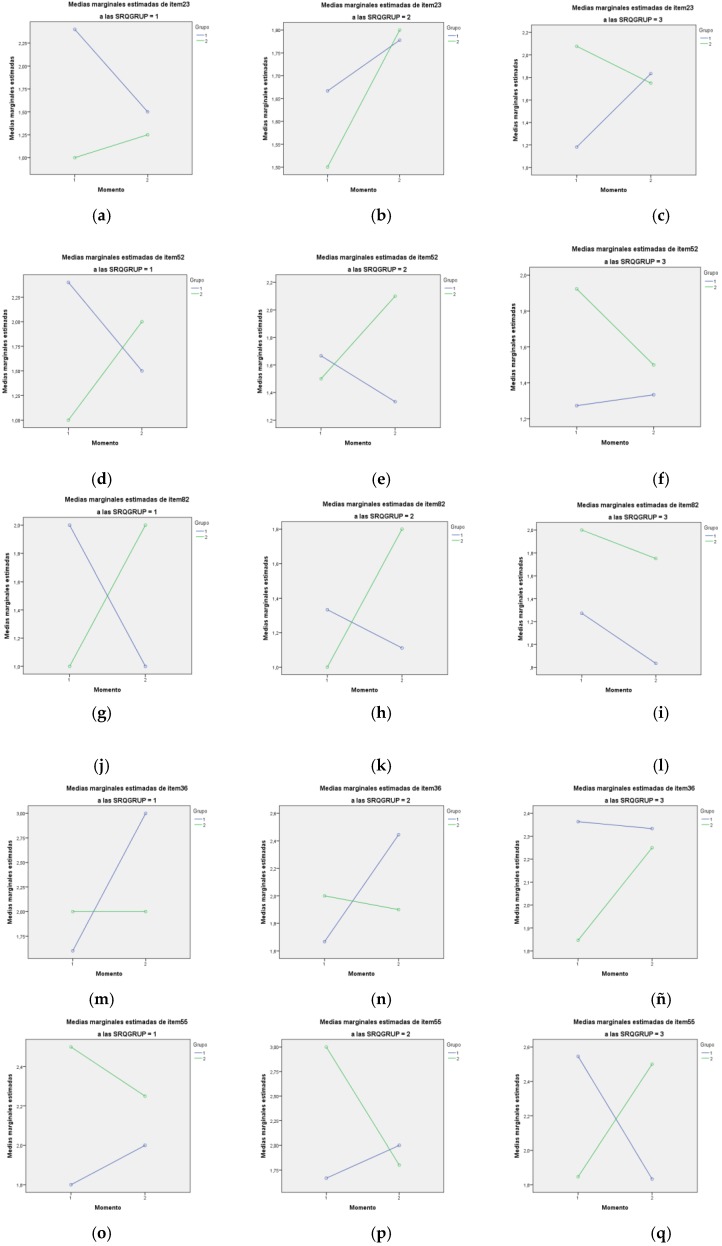
Interaction effect SR (SRQGRUP) × Tratament (GRUPs) × Times (Moment) on items 23, 52, and 82 (behavior directed toward emotion) and on items 20, 36, and 55 (behavior directed toward the problem). SR Group by level: 1—low; 2—medium; 3—high. Tratament: 1—Experimental; 2—Control. Key: Medias marginales estimadas—estimated marginal means; Momento—time: 1–2. (**a**,**d**,**g**,**j**,**m**,**o**) Effect on the low group of self-regulation; (**b**,**e**,**h**,**k**,**n**,**p**) Effect on the medium group of self-regulation; (**c**,**f**,**i**,**l**,**ñ**,**q**) Effect on the high group of self-regulation.

The multivariate analysis Self-Regulation Level × Treatment × Time showed a significant main effect of Self-Regulation Level, SR (F(94,28) = 1.696, *p* < 0.05, *n*^2^ = 0.891, power = 0.952) and of Treatment (F(47,13) = 5.427, *p* < 0.001, *n*^2^ = 0.952, power = 0.999). There were also significant partial effects on items of specific coping behaviors. In the case of emotion-focused coping, there was a significant partial effect on item 23 (F(2,70) = 3.129 (Pillai), *p* < 0.05, *n*^2^ = 0.090, power = 0.581), item 29 (F(2,70) = 3.408 (Pillai), *p* < 0.05, *n*^2^ = 0.104, power = 0.609), item 52 (F(2,62) = 4.396, *p* < 0.01, *n*^2^ =0.130, power = 0.739), and item 82 (F(2,62) = 3.318, *p* < 0.05, *n*^2^ = 0.101, power = 0.607). In problem-focused coping, there was a significant partial effect on item 20 (F(2,62) = 4.921, *p* < 0.001, *n*^2^ = 0.143, power = 0.897), item 36 (F(2,62) = 3.023, *p* < 0.056, *n*^2^ = 0.089, power = 0.586), and item 55 (F(2,62) = 3.730, *p* < 0.054, *n*^2^ = 0.107, power = 0.943). Specific values are presented in Table 5. Figure 5 graphically illustrates these effects on the items.

## 4. Discussion

These results suggest different analyses in light of the hypotheses. Hypothesis 1 suggested that students’ level of self-regulation would be interactive with mindfulness treatment or, in other words, that a higher level of self-regulation would be accompanied by a reduction in inadequate emotion-based strategies, an increase in adequate strategies, and maintenance of problem-focused strategies. In contrast, students with a lower level of self-regulation would increase their use of emotion-focused strategies, but not problem-focused strategies. 

This hypothesis was partially fulfilled. In the first place, a crossed effect appeared, self-regulation × time, for the total coping strategies score. This result shows that a greater number of coping strategies were produced at Time 2, especially when subjects had a higher level of regulation, at a time that they were facing greater stress and more behavioral requirements. We must remember that the second assessment took place during the final exam period in June. Moreover, this is consistent with prior evidence that had already established relationships between level of self-regulation and number of coping strategies used [52,53].

On the other hand, the evidence indicates that mindfulness treatment had no general effect of increasing the total number of coping strategies used, only specific types of strategies. In fact, the analysis by dimensions showed an interaction between level of self-regulation × mindfulness treatment, offering evidence that, with lower levels of self-regulation, mindfulness treatment led to a reduction in emotion-focused coping strategies. This result is consistent with prior evidence that has repeatedly shown greater self-regulation to be associated with greater use of problem-focused coping strategies and lesser use of emotion-focused strategies. Students who are more self-regulated encounter fewer stressful experiences; consequently, fewer strategies need to be applied for stress management [54,55].

More specifically, Hypothesis 2 expected a significant increase in coping behaviors typical of mindfulness, corresponding to students’ level of self-regulation and the treatment received. It seemed plausible that, with greater self-regulation, there would be a decrease in negative emotion-focused strategies (suppressing feelings, avoidance, and substance use), an increase in positive emotion-focused strategies (expressing feelings, positive attitude), and a greater number of problem-focused strategies (help-seeking and social support).

This result was corroborated in part, and differently according to the type of student. Students with a low level of self-regulation—who have more negative emotional experiences—must invest more effort and a greater number of emotion-focused coping strategies in order to manage stress [55,56]. This is verified more specifically in the factors referring to strategies of avoidant distraction and emotional venting and isolation, which are significantly reduced by mindfulness treatment in all cases, especially so in students with low self-regulation. This result is consistent with prior research reports [56].

The effects that are produced in problem-focused strategies have great interest because of the crossed effect. Thus, while university students who had medium or high levels of self-regulation increased their use of problem-focused strategies, students with low self-regulation reduced their use of these (actions directed at the causes, and positive reappraisal and firmness), although all groups significantly increased their use of self-talk. In other words, students who were low in self-regulation seemed to adopt a coping strategy of nonproductive effort, choosing to focus on better internal messages, unlike the medium- and high-level students who increased all their strategies. To proceed in this manner, while it may be adaptive in some cases, it does not appear to be a well-adjusted tendency; it is more of a self-protecting strategy (self-regulating) for minimizing the negative emotional factor, in which case it might be considered functional for the student.

Finally, analysis of discrete behaviors significantly confirms the hypothesis, because there was a crossed effect of reduction in expected behaviors, in increasing proportion with lower levels of self-regulation, for the behaviors of preparing for the worst, keeping feelings to oneself (a-regulatory strategies) and drinking, smoking and isolating oneself, to cope with the problem (dysregulatory strategies). This result is especially relevant for preventive intervention, because such behaviors—characteristic of university students with low regulation—have already been revealed as the link between academic stress and health problems [56]. On the other hand, the evidence shows an increase in discrete, emotion-focused coping behaviors, typical of a positive approach to academic problems, such as looking at problems in a positive light, not paying attention to negative feelings when making a decision, and asking for help (self-regulating strategies). Prior evidence has revealed the goodness of these behaviors for coping positively with problems of daily life, and they are constituents of resilience [43,57].

This research study has several important limitations to be considered. On one hand, the sample itself, including only university students, is one bias that should be recognized. On the other hand, gender differences have not been addressed, although prior research has shown significant differences in use of coping strategies according to this variable [58]. Finally, students were not identified as being self-regulatory, a-regulatory, or dysregulatory [2]. Consequently, these aspects should be analyzed in future research in order to determine whether the effects that appeared here are invariable in nature.

In any case, the question for future research should not be whether this technique is effective or not, but rather, what type of personalized, differential treatment in mindfulness is needed for each type of student for economizing time and effort, having taken into account the different effects that are found. Personalized treatment—suited to each person—should begin to find its way into these types of interventions, not unlike what is already common in other fields of healthcare intervention.

## 5. Conclusions

These results show a differential panorama of how students who are low-medium-high in self-regulation benefit from mindfulness. We observe that the low-level self-regulators showed a reduction in the number of coping strategies, both maladjusted emotion-focused strategies and well-adjusted problem-focused strategies, while the medium- and high-level self-regulators, especially, are able to reduce inadequate emotion-focused strategies and increase problem-focused strategies. Level of self-regulation may be considered to have a clear mediating or buffering effect on treatment in the line of study referred to as person × treatment interaction [59] and of the Theory of Self- vs. External-Regulation [2]. This evidence recommends the analysis of differential, interactive effects that are produced by students’ level of self-regulation and the intervention program. Beyond the general effects, it is essential to pay attention to the specific effects on each type of student in order to better adjust the program to these characteristics.

In any event, these results further extend the already plentiful evidence that mindfulness as a training technique contributes to substantial improvement in coping strategies, being in itself a technique that makes many possibilities available to the subject for understanding and strategic regulation of his or her emotional experiences [42,43,60,61,62].

With respect to the mechanisms or variables that are responsible for the changes that are produced by MBIs, they may intervene positively in a variety of ways. For example, acceptance, exposure, and emotional self-control are primary behavioral mechanisms for explaining the effects of mindfulness [14]. More recently, other mechanisms that have been proposed are psychological, emotional, and behavioral flexibility, neurological and physiological changes, or, for example, decentering, desautomatization, and detachment processes. For more details, see [25,26]. These mechanisms help persons to be more aware of their experiences and internal processes, to regulate their emotions, and to develop healthier, more effective behaviors, thus producing more adaptive, positive coping strategies.

## Figures and Tables

**Chart 1:** Types of Coping Strategies and Sample Items from the Short EEC (Coping Strategies Questionnaire) version.

*Emotion-focused coping (D1)*	*Example items*
F1. Avoidant distraction	I get away and forget the problem temporarily (change of environment)
F7. Reducing anxiety and avoidance	I practice some kind of sport in order to reduce my anxiety or tension
F8. Preparing for the worst	I prepare myself for the worst
F9. Emotional venting and isolation	I act irritable and aggressive toward others
F11. Resigned acceptance	I accept the problem as it is, since I cannot do anything to solve it
*Problem-focused coping (D2)*	
F2. Seeking family help and counsel	I talk with people I know who can do something to solve my problem
F5. Self-talk	I lay out a plan of action and try to carry it out
F10. Positive reappraisal and firmness	I try to see positive aspects of the situation
F12. Communicating feelings and social support	I feel better if I explain my problem to friends or family members
F13. Seeking alternative reinforcement	I start new activities (studies, etc.)

**Chart 2:** Summary of the Intervention Program.

Session	Exercises	Metaphors
**1**	* Introduction of all group members; * Sharing motives for participating in the meditation course and expectations; * Presentation of prerequisite conditions for beginning to meditate and different aspects to take into account when practicing meditation; * Explanation of the flow meditation practice; * Realization of the first meditation practice	
**2**	* Exercise of trying not to blink for one minute; * Finding analogies between the above exercise and control of one’s private events; * Meditation practice	Bamboo metaphor
**3**	* Exercise of observing sensations in the facial area * Meditation practice	River, Dirty glass, Operation, and Painful childbirth metaphors
**4**	* Exercise: observing sensations in the chest and abdominal area; * Meditation practice	Monkey and banana metaphors
**5**	* Exercise of observing sensations in the back area; * Exercise of remembering words; * Exercise of observing one’s thoughts; * Exercises: *Which numbers are they?* and *What’s your mother’s name?* * Meditation practice	The two monks metaphor
**6**	* Exercise of observing sensations in one’s arms; * Exercise of counting thoughts; * Meditation practice	Story of the two rings
**7**	* Exercise of observing sensations in one’s legs; * Meditation practice	Metaphor of the radio turned on
**8**	* Exercise of observing sensations throughout one’s body; * Exercise of analyzing the “I should’s” and “I ought to’s”	
**9**	* Exercise of accepting bothersome and inconvenient private events; * Exercise of saying yes and doing no, and saying no and doing yes; * Meditation practice	The computer and the polygraph metaphor, the Story of Miaou
**10**	* Meditation practice	Metaphor of the Indians collecting firewood

**Note:** Metaphors are types of analogies. An analogy is a comparison between two things to show their similarities or relationship. Metaphors are used to help people get distance from their thoughts and play an important role in Acceptance and Commitment Therapy (ACT).

**Table 1:** Interdependence relations between Self-regulation level (SR: L,M,H), Mindfulness treatment (Group 1,2), and time (Momento 1,2) on total coping strategies (*n* = 71).

Treatment	Time	Self-Regulation Level			
		1. Low (*n* = 13)	2. Medium (*n* = 24)	3. High (*n* = 34)	Total
1. Experimental	1 (*n* = 38)	2.01 (0.05)	1.60 (0.08)	1.58 (0.13)	1.70 (0.21)
	2 (*n* = 36)	1.79 (0.21)	1.73 (0.19)	1.67 (0.13)	1.72 (0.16)
	Total	1.95 (0.14)	1.70 (0.17)	1.61 (0.13)	1.71 (0.19)
2. Control	1 (*n* = 38)	1.58 (0.22)	1.69 (0.21)	1.66 (0.15)	1.65 (0.16)
	2 (*n* = 36)	1.71 (0.07)	1.77 (0.21)	1.71 (0.13)	1.74 (0.17)
	Total	1.67 (0.13)	1.75 (0.20)	1.67 (0.15)	1.70 (0.17)
Total	1 (*n* = 38)	1.88 (0.23)	1.64 (0.13)	1.62 (0.15)	1.68 (0.19)
	2 (*n* = 36)	1.74 (0.11)	1.75 (0.20)	1.69 (0.12)	1.73 (0.16)
	Total	1.82 (0.19)	1.73 (0.19)	1.64 (0.14)	1.70 (0.18)

**Table 2:** Interdependence relations between *Self-Regulation Level, Mindfulness Treatment* and *Time*, in dimensions of coping strategies.

Treatment	Time	Self-Regulation Level			
		1. Low (*n* = 13)	2. Medium (*n* = 24)	3. High (*n* = 34)	Total
1. Experimental		*Dimension 1.*	*Emotion-focused*	*coping*	
	1 (*n* = 38)	2.04 (0.13)	1.62 (0.17)	1.52 (0.22)	1.68 (0.29)
	2 (*n* = 36)	1.71 (0.19)	1.68 (0.18)	1.45 (0.39)	1.60 (0.19)
	Total	1.95 (0.21)	1.66 (0.17)	1.45 (0.32)	1.64 (0.25)
2. Control	1 (*n* = 38)	1.67 (0.27)	1.82 (0.36)	1.60 (0.17)	1.63 (0.19)
	2 (*n* = 36)	1.65 (0.11)	1.78 (0.17)	1.67 (0.17)	1.72 (0.16)
	Total	1.66 (0.15)	1.78 (0.18)	1.62 (0.17)	1.68 (0.18)
Total	1 (*n* = 38)	1.94 (0.24)	1.70 (0.21)	1.56 (0.19)	1.65 (0.24)
	2 (*n* = 36)	1.67 (0.12)	1.73 (0.18)	1.54 (0.19)	1.66 (0.19)
	Total	1.81 (0.23)	1.72 (0.18)	1.56 (0.19)	1.66 (0.22)
1. Experimental		*Dimension 2.*	*Problem-focused*	*coping*	
	1 (*n* = 38)	1.97 (0.15)	1.58 (0.07)	1.64 (0.15)	1.72 (0.20)
	2 (*n* = 36)	1.88 (0.23)	1.78 (0.26)	1.90 (0.18)	1.84 (0.22)
	Total	1.95 (0.16)	1.73 (0.24)	1.73 (0.20)	1.77 (0.22)
2. Control	1 (*n* = 38)	1.49 (0.18)	1.56 (0.16)	1.72 (0.17)	1.67 (0.18)
	2 (*n* = 36)	1.78 (0.03)	1.76 (0.31)	1.74 (0.10)	1.76 (0.23)
	Total	1.68 (0.17)	1.72 (0.29)	1.73 (0.15)	1.72 (0.21)
Total	1 (*n* = 38)	1.83 (0.27)	1.58 (0.09)	1.68 (0.16)	1.70 (0.19)
	2 (*n* = 36)	1.81 (0.12)	1.77 (0.28)	1.84 (0.16)	1.80 (0.23)
	Total	1.82 (0.21)	1.73 (0.26)	1.73 (0.17)	1.75 (0.21)

**Table 3:** Interdependence relations between Self-Regulation level, Mindfulness Treatment and Time, in emotion-based factors of coping strategies.

Treatment	Time	Self-Regulation Level			
		1. Low (*n* = 13)	2. Medium (*n* = 24)	3. High (*n* = 34)	Total
1. Experimental		*Factor 1.*	*Avoidant*	*distraction*	
	1 (*n* = 38)	1.66 (0.00)	1.44 (0.38)	1.87 (0.22)	1.75 (0.26)
	2 (*n* = 36)	1.50 (0.23)	1.81 (0.44)	1.44 (0.27)	1.64 (0.39)
	Total	1.61 (0.12)	1.72 (0.44)	1.72 (0.31)	1.70 (0.33)
2. Control	1 (*n* = 38)	2.00 (0.47)	2.50 (0.23)	1.71 (0.44)	1.84 (0.48)
	2 (*n* = 36)	1.66 (0.86)	1.80 (0.23)	1.66 (0.27)	1.74 (0.42)
	Total	1.77 (0.72)	1.91 (0.35)	1.70 (0.40)	1.79 (0.45)
Total	1 (*n* = 38)	1.76 (0.25)	1.86 (0.64)	1.79 (0.36)	1.79 (0.38)
	2 (*n* = 36)	1.61 (0.68)	1.80 (0.33)	1.53 (0.28)	1.69 (0.40)
	Total	1.69 (0.48)	1.81 (0.40)	1.71 (0.35)	1.74 (0.39)
1. Experimental		*Factor 2.*	*Emotional venting*	*and isolation*	
	1 (*n* = 38)	1.96 (0.26)	1.53 (0.41)	1.34 (0.35)	1.53 (0.41)
	2 (*n* = 36)	1.84 (0.02)	1.37 (0.44)	1.33 (0.39)	1.36 (0.38)
	Total	1.80 (0.34)	1.41 (0.42)	1.34 (0.35)	1.45 (0.40)
2. Control	1 (*n* = 38)	1.70 (0.14)	1.30 (0.14)	1.75 (0.36)	1.69 (0.35)
	2 (*n* = 36)	1.85 (0.19)	1.70 (0.22)	1.55 (0.19)	1.73 (0.22)
	Total	1.80 (0.17)	1.68 (0.27)	1.70 (0.33)	1.71 (0.29)
Total	1 (*n* = 38)	1.88 (0.25)	1.44 (0.32)	1.56 (0.41)	1.61 (0.39)
	2 (*n* = 36)	1.70 (0.27)	1.57 (0.38)	1.42 (0.33)	1.55 (0.36)
	Total	1.80 (0.27)	1.55 (0.37)	1.52 (0.39)	1.58 (0.37)

**Table 4:** Interdependence relations between Self-Regulation level, Mindfulness Treatment and Time, in Problem-based Factors of Coping.

Treatment	Time	Self-Regulation Level			
		1. Low (*n* = 13)	2. Medium (*n* = 24)	3. High (*n* = 34)	Total
1. Experimental		*Factor 3.*	*Actions directed at*	*causes*	
	1 (*n* = 38)	1.86 (0.18)	1.66 (0.57)	1.24 (0.42)	1.47 (0.47)
	2 (*n* = 36)	1.33 (0.47)	1.37 (0.48)	1.72 (0.25)	1.49 (0.42)
	Total	1.71 (0.35)	1.44 (0.49)	1.41 (0.43)	1.48 (0.44)
2. Control	1 (*n* = 38)	1.16 (0.23)	1.50 (0.70)	1.76 (0.45)	1.66 (0.48)
	2 (*n* = 36)	1.66 (0.27)	1.70 (0.45)	1.75 (0.63)	1.70 (0.44)
	Total	1.50 (0.34)	1.66 (0.47)	1.76 (0.48)	1.68 (0.45)
Total	1 (*n* = 38)	1.66 (0.38)	1.60 (0.54)	1.52 (0.50)	1.56 (0.48)
	2 (*n* = 36)	1.55 (0.34)	1.54 (0.48)	1.73 (0.40)	1.60 (0.44)
	Total	1.61 (0.35)	1.55 (0.48)	1.58 (0.48)	1.58 (0.46)
1. Experimental		*Factor 5.*	*Self-Talk*		
	1 (*n* = 38)	1.70 (0.44)	1.83 (0.76)	2.27 (0.41)	2.05 (0.52)
	2 (*n* = 36)	3.00 (0.00)	2.44 (0.63)	2.25 (0.52)	2.44 (0.58)
	Total	2.07 (0.73)	2.29 (0.68)	2.26 (0.43)	2.23 (0.57)
2. Control	1 (*n* = 38)	2.25 (0.35)	2.00 (0.12)	1.80 (0.38)	1.88 (0.37)
	2 (*n* = 36)	2.12 (0.25)	1.80 (0.53)	2.12 (0.27)	1.94 (0.45)
	Total	1.77 (0.72)	1.91 (0.35)	1.70 (0.40)	1.79 (0.45)
Total	1 (*n* = 38)	1.85 (0.47)	1.90 (0.54)	2.02 (0.45)	1.97 (0.42)
	2 (*n* = 36)	2.41 (0.49)	2.10 (0.65)	2.20 (0.42)	2.18 (0.56)
	Total	2.11 (0.54)	2.06 (0.63)	2.07 (0.44)	2.07 (0.52)
1. Experimental		*Factor 10.*	*Positive reappraissal*	*and firmness*	
	1 (*n* = 38)	2.40 (0.54)	1.33 (0.57)	1.09 (0.53)	1.47 (0.77)
	2 (*n* = 36)	1.50 (0.70)	1.66 (0.86)	1.83 (0.75)	1.70 (0.77)
	Total	2.14 (0.69)	1.58 (0.79)	1.35 (0.70)	1.58 (0.76)
2. Control	1 (*n* = 38)	1.00 (0.10)	1.00 (0.10)	1.69 (0.75)	1.52 (0.71)
	2 (*n* = 36)	1.75 (0.50)	1.90 (0.73)	1.25 (0.50)	1.72 (0.66)
	Total	1.50 (0.54)	1.75 (0.75)	1.58 (0.71)	1.62 (0.68)
Total	1 (*n* = 38)	2.00 (0.81)	1.20 (0.44)	1.41 (0.71)	1.50 (0.73)
	2 (*n* = 36)	1.66 (0.51)	1.78 (0.78)	1.60 (0.69)	1.71 (0.71)
	Total	1.84 (0.68)	1.66 (0.76)	1.47 (0.70)	1.60 (0.72)

**Table 5:** Interdependence relations between Mindfulness Treatment, levels of Self-Regulation in specific emotion-focused coping behaviors.

Treatment	Time	Self-Regulation Level			
		1. Low (*n* = 13)	2. Medium (*n* = 24)	3. High (*n* = 34)	Total
1. Experimental		*Item 23 (emotion)*	*I prepare for the*	*worst*	
	1 (*n* = 38)	2.40 (0.54)	1.67 (0.57)	1.18 (0.40)	2.00 (0.81)
	2 (*n* = 36)	1.50 (0.70)	1.78 (0.83)	1.83 (0.75)	1.33 (0.51)
	Total	2.14 (0.69)	1.75 (0.75)	1.41 (0.61)	1.69 (0.75)
2. Control	1 (*n* = 38)	1.00 (0.00)	1.50 (0.70)	2.08 (0.49)	1.60 (0.54)
	2 (*n* = 36)	1.25 (0.50)	1.80 (0.78)	1.75 (0.50)	1.69 (0.78)
	Total	1.17 (0.40)	1.75 (0.75)	2.00 (0.28)	1.75 (0.73)
Total	1 (*n* = 38)	1.58 (0.69)	1.88 (0.60)	1.72 (0.65)	1.72 (0.65)
	2 (*n* = 36)	1.76 (0.75)	1.67 (0.68)	1.71 (0.71)	1.71 (0.71)
	Total	1.67 (0.71)	1.77 (0.64)	1.72 (0.68)	1.72 (0.68)
1. Experimental		*Item 29 (emotion)*	*I criticize and scold*	*myself in these*	*situations*
	1 (*n* = 38)	1.80 (0.44)	2.33 (0.57)	1.27 (0.46)	1.58 (0.60)
	2 (*n* = 36)	1.00 (0.10)	1.11 (0.78)	1.17 (0.75)	1.12 (0.69)
	Total	1.57 (0.53)	1.42 (0.90)	1.24 (0.56)	1.36 (0.68)
2. Control	1 (*n* = 38)	1.00 (0.00)	1.50 (0.70)	2.08 (0.76)	1.58 (0.60)
	2 (*n* = 36)	1.50 (0.57)	1.80 (0.78)	1.50 (0.57)	1.83 (0.34)
	Total	1.33 (0.56)	1.75 (0.75)	1.59 (0.74)	1.77 (0.63)
Total	1 (*n* = 38)	1.57 (0.53)	2.00 (0.54)	1.71 (0.75)	1.72 (0.70)
	2 (*n* = 36)	1.33 (0.51)	1.47 (0.84)	1.30 (0.65)	1.40 (0.73)
	Total	1.44 (0.51)	1.58 (0.83)	1.59 (0.74)	1.56 (0.73)
1. Experimental		*Item 52 (emotion)*	*I try to*	*keep my feelings*	*to myself*
	1 (*n* = 38)	2.40 (0.89)	1.67 (0.55)	1.27 (0.46)	1.63 (0.76)
	2 (*n* = 36)	1.50 (0.70)	1.33 (0.50)	1.33 (0.80)	1.35 (0.60)
	Total	2.14 (0.90)	1.42 (0.15)	1.29 (0.58)	1.50 (0.69)
2. Control	1 (*n* = 38)	1.00 (0.10)	1.50 (0.70)	1.92 (0.49)	1.76 (0.52)
	2 (*n* = 36)	2.00 (0.00)	2.10 (0.73)	1.50 (0.57)	1.94 (0.63)
	Total	1.66 (0.51)	2.00 (0.73)	1.82 (0.52)	1.83 (0.60)
Total	1 (*n* = 38)	2.00 (1.00)	1.60 (0.54)	1.63 (0.57)	1.69 (0.68)
	2 (*n* = 36)	1.83 (0.48)	1.74 (0.73)	1.75 (0.50)	1.66 (0.68)
	Total	1.92 (0.76)	1.71 (0.46)	1.82 (0.52)	1.78 (0.61)
1. Experimental		*Item 82 (emotion)*	*I try to feel better by*	*eating, smoking*	*or taking*
	1 (*n* = 38)	2.00 (0.00)	1.33 (0.57)	1.27 (0.46)	1.47 (0.51)
	2 (*n* = 36)	0.66 (0.57)	1.00 (0.66)	0.83 (0.40)	0.89 (0.56)
	Total	1.50 (0.75)	1.07 (0.64)	1.11 (0.48)	1.18 (0.60)
2. Control	1 (*n* = 38)	1.00 (0.10)	1.00 (0.10)	2.00 (0.55)	1.77 (0.64)
	2 (*n* = 36)	2.00 (0.81)	1.80 (0.42)	1.75 (0.50)	1.83 (0.51)
	Total	1.66 (0.81)	1.66 (0.49)	1.94 (0.53)	1.48 (0.66)
Total	1 (*n* = 38)	1.00 (0.00)	1.50 (0.74)	2.07 (0.47)	1.66 (0.30)
	2 (*n* = 36)	1.25 (0.50)	1.80 (0.78)	1.75 (0.50)	1.83 (0.34)
	Total	1.16 (0.27)	1.75 (0.43)	2.00 (0.28)	1.77 (0.63)
1. Experimental		*Item 20 (problem)*	*When things go*	*rough put a*	*brave face*
	1 (*n* = 38)	2.40 (0.54)	1.33 (0.57)	1.09 (0.53)	1.47 (0.77)
	2 (*n* = 36)	1.50 (0.70)	1.67 (0.86)	1.83 (0.75)	1.71 (0.77)
	Total	2.14 (0.69)	1.68 (0.73)	1.35 (0.70)	1.58 (0.76)
2. Control	1 (*n* = 38)	1.00 (0.10)	1.00 (0.10)	1.69 (0.75)	1.53 (0.71)
	2 (*n* = 36)	1.75 (0.50)	1.90 (0.73)	1.25 (0.50)	1.72 (0.66)
	Total	1.50 (0.54)	1.75 (0.76)	1.59 (0.75)	1.63 (0.69)
Total	1 (*n* = 38)	2.00 (0.81)	1.50 (0.70)	1.42 (0.71)	1.50 (0.73)
	2 (*n* = 36)	1.67 (0.15)	1.80 (0.78)	1.60 (0.69)	1.71 (0.71)
	Total	1.65 (0.69)	1.75 (0.43)	1.47 (0.70)	1.61 (0.72)
1. Experimental		*Item 36 (emotion)*	*I try to not let*	*keep feelings*	*Interferen.*
	1 (*n* = 38)	1.60 (0.54)	1.67 (0.57)	2.36 (0.50)	2.05 (0.62)
	2 (*n* = 36)	3.00 (0.00)	2.44 (0.72)	2.33 (0.51)	2.47 (0.61)
	Total	2.00 (0.86)	2.25 (0.75)	2.35 (0.49)	2.25 (0.64)
2. Control	1 (*n* = 38)	2.00 (0.00)	2.00 (0.23)	1.85 (0.55)	1.88 (0.48)
	2 (*n* = 36)	2.00 (0.00)	1.90 (0.78)	2.25 (0.50)	2.00 (0.59)
	Total	2.00 (0.00)	1.92 (0.66)	2.15 (0.55)	1.94 (0.53)
Total	1 (*n* = 38)	1.71 (0.48)	1.80 (0.44)	1.97 (0.56)	1.97 (0.56)
	2 (*n* = 36)	2.00 (0.55)	2.08 (0.71)	2.23 (0.64)	2.23 (0.64)
	Total	2.00 (0.55)	2.08 (0.71)	2.10 (0.63)	2.10 (0.63)
1. Experimental		*Item 55 (emotion)*	*I seek*	*professional*	*help*
	1 (*n* = 38)	1.80 (0.44)	1.67 (0.57)	2.55 (0.52)	2.40 (0.54)
	2 (*n* = 36)	2.00 (0.00)	2.00 (0.70)	1.83 (0.40)	1.50 (0.70)
	Total	1.86 (0.37)	1.92 (0.67)	2.29 (0.58)	2.14 (0.69)
2. Control	1 (*n* = 38)	2.50 (0.70)	3.00 (0.00)	1.92 (0.55)	1.50 (0.70)
	2 (*n* = 36)	2.25 (0.50)	1.80 (0.78)	2.50 (0.57)	1.50 (0.57)
	Total	2.33 (0.51)	2.00 (0.85)	2.00 (0.61)	1.50 (0.54)
Total	1 (*n* = 38)	2.00 (0.00)	2.20 (0.87)	2.20 (0.87)	2.14 (0.63)
	2 (*n* = 36)	2.17 (0.48)	1.89 (0.73)	1.89 (0.73)	1.50 (0.64)
	Total	2.08 (0.49)	1.96 (0.71)	1.76 (0.75)	1.85 (0.64)
